# The Costs of Confronting Osteoporosis: Cost Study of an Australian Fracture Liaison Service

**DOI:** 10.1002/jbm4.10046

**Published:** 2018-04-18

**Authors:** Gabor Major, Rod Ling, Andrew Searles, Fiona Niddrie, Ayano Kelly, Elizabeth Holliday, John Attia, Nikolai Bogduk

**Affiliations:** ^1^ Bone and Joint Centre John Hunter Hospital New Lambton Australia; ^2^ University of Newcastle Faculty of Health and Medicine Callaghan Australia; ^3^ Hunter Medical Research Institute (HMRI) New Lambton Heights Australia; ^4^ Canberra Hospital Rheumatology Department Woden Australia

**Keywords:** FRACTURE LIAISON CLINIC, FRAGILITY FRACTURE, REFRACTURE, OSTEOPOROSIS, HEALTH ECONOMICS

## Abstract

Fracture liaison services (FLS) are an accepted approach to lowering rates of osteoporotic refractures. However, resource allocations to FLS are open to challenge, as most relevant cost analyses are based on anticipated, rather than observed, benefits. To support informed decision making, we have estimated the cost of operating an FLS, from the perspective of the Australian health system, with real life costs. On the basis of hospital records, we compared total costs of two cohorts of patients presenting with minimal trauma fractures (MTFs) at two hospital emergency departments (EDs) across a 6‐month period (July to December 2010). The treatment cohort (FLS Cohort, *n* = 515) attended an ED at a hospital offering FLS post‐fracture care; the Usual Care Cohort (*n* = 416) attended an ED at a hospital without an FLS. Hospital records were reviewed for further attendance of both groups at their respective hospitals’ EDs with refractures for the subsequent 3 years. Costs were constructed from “bottom up” with a “microcosting” approach. Total costs for both cohorts included any FLS and the costs of refractures. Cohort costs were estimated for every 1000 patients over the 3 observed years. Compared with the Usual Care Cohort, the FLS Cohort had 62 fewer fractures per 1000 patients and $617,275 lower costs over 3 years. In a sensitivity analysis, where 20% of the Usual Care Cohort received FLS preventative treatment, FLS Cohort costs were lower by $880,154. As both hospitals consistently process around 2000 patients per year, the estimated annual saving is $1.2 million to $1.8 million (Australian dollars). From the perspective of the Australian public health system, investment in FLS can be a financially effective way of reducing the cost of osteoporotic fracture management. © 2018 The Authors *JBMR Plus* published by Wiley Periodicals, Inc. on behalf of American Society for Bone and Mineral Research.

## Introduction

The worldwide increase in the incidence of fragility fractures has been described as a coming “tsunami” that poses a significant threat to health budgets.^1^ In Australia during 2012, the total direct cost of fractures associated with osteoporosis and osteopenia for people older than 50 years was $1.76 billion (Australian dollars [AUD]).^2^ The problem is exacerbated by a worldwide deficiency in treatment delivery, wherein only 10% to 20% of patients at the highest risk of fracture are treated.^3^ To address the problem, organizations such as the International Osteoporosis Foundation and the American Society for Bone and Mineral Research have issued position papers calling for the development and implementation of fracture liaison services (FLS) as the most effective way to address this challenge.^3^, ^4^, ^5^

This strategy is based on the recognition that a person who suffers a fracture after minor injury—generally referred to as a minimal trauma fracture (MTF)—is at high risk of further fractures,^6^ warranting prompt evaluation and treatment with measures that are effective and safe. Although the initial acceptance of this approach was based on projected benefits, the effectiveness of FLS has now also been confirmed by observed reductions in refracture rates.^7^, ^8^, ^9^ A recent audit of FLS in England estimated that if all fracture patients received an FLS consultation, almost 22,000 refractures would be avoided within 5 years at a gross saving just on hip fractures of more than £151 million.^10^

Because prior evaluations have been based on projected or anticipated benefits rather than observed data, there has been uncertainty around the true cost and effect size from FLS. Given the relevance of MTFs to public health, this uncertainty requires resolving through further research on both the effectiveness and cost of FLS.

**Figure 1:**
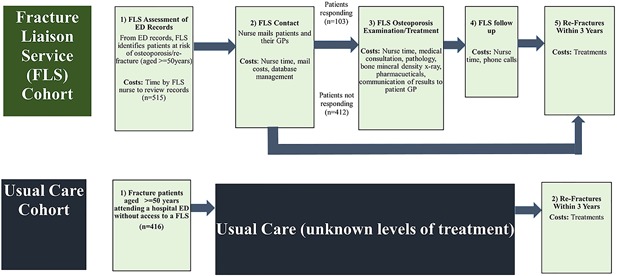
Care paths: fracture liaison service and usual care cohorts.

The John Hunter Hospital (JHH) in New South Wales (NSW), Australia, maintains a specialized FLS, which assesses all patients aged 50 years and older who present to the JHH emergency department (ED) with a fracture. All patients with a MTF are contacted. Excluding those who were started on anti‐osteoporotic treatment during their hospitalization, living out of area, or discharged to a palliative‐ or aged‐care facility, the remainder are offered review within a specialized clinic. This service provides clinical assessment, bone health education, relevant investigations (blood tests and bone densitometry), and treatment appropriate to the individual patient. The findings and recommendations about treatments including anti‐osteoporosis drugs, are communicated to the patient's family doctor (Fig. 1).

At hospitals without FLS, there are no defined pathways for post‐fracture management or advice. Although it is likely that some MTF patients presenting at the EDs of these hospitals receive management, the percentages involved and their types of interventions are not specifically known. In a previous study comparing JHH FLS patients and patients receiving Usual Care, the risk of major refracture in the JHH group was lower by 40%, and of any refracture, by 30%. Over 3 years, one new refracture is prevented for 20 patients processed by the FLS.^9^

The present study was undertaken as a follow‐up costing exercise to provide directly observed evidence to decision makers about FLS implementation. We conducted a micro‐costing exercise of all components of an FLS. We also measured and compared the refracture rates of both cohorts. To our knowledge, this study is the only micro‐costing analysis of an FLS.

## Materials and Methods

### Study design

This study takes the perspective of Australian public health services and uses patient record data from our previous study^9^ approved by the Hunter New England Human Research Ethics committee. The perspective of public health services was used because it was intended to capture costs for the largely state‐funded local health districts as well as federally funded services such as the Medical Benefits Scheme (MBS) and Pharmaceutical Benefits Scheme (PBS). The study has a time horizon of 3 years.

This is a historical cohort study of two non‐randomly chosen comparator groups, concurrently observed over 3 years. The treatment cohort, or FLS Cohort, presented at JHH ED with MTFs. All patients were contacted and eligible patients offered a review appointment at a specialist clinic at no personal cost to themselves. The control cohort presented to the ED of another NSW hospital—comparable in bed size, function, and catchment^9^—which had no FLS. Hereafter, this group will be referred to as the Usual Care Cohort. Patients in both cohorts were aged 50 and older and presented at their respective EDs over the 6 months from July to December 2010.

The data also describe cohort members’ subsequent presentations at EDs for refracture treatments over the 3 years after their initial presentations. Further description of patient data was previously reported by Nakayama and colleagues.^9^

Costs were gathered for FLS administration, investigations, and treatments. Costing took the “bottom up,” or micro‐costing approach, where all contributing costs were identified, gathered, and aggregated.

A cost model was constructed in a Microsoft Excel 2013 workbook in three stages. The key stages in the post‐fracture pathways of the FLS and Usual Care Cohorts were identified. Successive points in the FLS pathway were: 1) FLS assessment of ED records; 2) FLS mail contact; 3) FLS clinic assessment and treatment; 4) FLS follow‐up telephone calls; and 5) refractures within the subsequent 3 years. The Usual Care pathway had only one post‐fracture stage, that being refractures within 3 years (Fig. 1).

For each stage, costs were identified, gathered, entered into an Excel workbook, aggregated, and divided by numbers of cohort patients to give total costs “per cohort patient.” Base populations for calculations of “per patient costs” include all patients in the respective cohorts, including those in the FLS Cohort who did not accept the offer to attend an FLS clinic. As undertaken in similar studies,^(11,12)^ total per cohort patient costs for all stages were converted to allow for direct cohort comparisons. Costs per processed fracture patient were calculated for both cohorts. For the FLS cohort, the calculation included all patients contacted for an appointment, including those who did not attend. Further, this study reports results in a common base of “per 1000 patients” (cost per patient multiplied by 1000).

Cost differences between the cohorts were calculated. As appropriate in studies of resource use, refracture incidence is calculated for both cohorts, using the total cohort populations as denominators and numbers of refractures for numerators (clinical incidence).^13^ A test for significant difference in refracture incidence between the two cohorts was run using Poisson regression with STATA (version 14.2) statistical software.^14^

### Costs and service use

Labor costs were determined with reference to the published NSW state wages award^15^, ^16^ for the pay level at which the JHH FLS nurses are hired (Clinical Nurse Specialist grade 2). FLS nurses identified their time commitments associated with each FLS stage. Labor costs for FLS medical consultants were assumed to be included in Medical Benefit Scheme (MBS) consultation fees. Overhead costs for the FLS office and consulting room (eg, electricity, computers, furniture, etc.) were calculated at 27.5% of labor cost as advised by JHH finance staff,^17^ who also gave information on postage and stationery costs. Telephone costs were based on advertised prices of Telstra,^18^ the Australian national telephone service provider, and the estimates of call numbers by FLS staff.^19^

Patient management costs were based on medical consultation charges as per the MBS items 110 (initial consultation by a consultant physician) and 116 (follow‐up consultation by consultant physician).^20^ We also costed investigations (bone mineral density and a pathology panel: full blood count, calcium, vitamin D, kidney and liver function tests, thyroid and parathyroid hormone) as per MBS items: 12306, 65070, 66512, 66833, 66716, and 66695.^20^ The list of investigations and their appropriate MBS codes was supplied by FLS staff. Estimations of the number of repeat FLS consultations were based on data used by Nakayama and colleagues.^9^

For specific anti‐osteoporosis therapies, there were no record‐level data on the types of therapies prescribed to individual patients. However, based on the findings of an earlier study^21^ at the JHH FLS, approximately 66% of patients were prescribed specific anti‐osteoporosis therapy. The drug therapies prescribed by the clinic are: risedronate, denosumab, alendronate, zoledronic acid, and teriparatide. Across these therapies, a weighted average 3‐year cost per treated patient was calculated. Item costs were gathered from the Pharmaceutical Benefits Scheme (PBS) website.^22^ For the cost per treated patient, we averaged the costs, weighting each by the relative level of prescriptions in 2016, as downloaded from the Department of Human Services website.^23^ Our estimated cost per treated person over 3 years was AUD $1416.

Refracture treatment costs were estimated using results from a major Australian study.^2^ First, we categorized refractures of both cohorts according to the scheme used by Watts and colleagues:^2^ hip, vertebral, wrist, and other. We then estimated average expected cost for 2015 in each category, dividing Watts and colleagues’^2^ estimates of national refracture numbers by their estimates of associated total direct costs. Refracture cost estimates included hospitalization and ambulance use, bisphosphonate therapy, pathology tests, and general practitioner visits. Last, we applied these average costs to the refractures of each cohort.

All financial data and results are quoted in 2015–16 Australian dollars. Where necessary, inflation of cost values was conducted with reference to the Total Health Price Index and Industry Wide Index.^24^ All reported cost savings reflect economic opportunity cost, *not* accounting cost (see Table 1 for a summary of costs).

**Table 1:** Costs Summary: Fracture Liaison Service (FLS) and Usual Care Cohorts

FLS Cohort						
	No.					Source [reference no.]
Patients						
JHH ED MTF patients contacted by JHH FLS	515					9
Attended FLS clinic (20%)	103					9

	Cost per unit (AUD)	Units utilized (*n*)	Total cost (AUD)	Cost per patient contacted (AUD)	Cost per 1000 patients contacted (AUD)	Source [reference no.]
Labor						
Nursing staff	$47,406	1.3	$61,628	$120	$119,666	15, 16
Office/stationery costs						
Paper	$0.00096	2575	$2.46	$0.005	$5	9
Envelopes	$0.15	2060	$300	$0.58	$583	9
Postage	$1.00	2060	$2060	$4.00	$4000	9
Follow‐up phone calls	$0.22	309	$68	$0.13	$132	18, 19
Consultations, tests, medications						
Initial consultation: MBS 110	$150.90	103	$15,543	$30	$30,180	20
Follow‐up: MBS 116	$75.50	57	$4304	$8	$8356	19, 20
Bone mineral density: MBS 12306	$102.40	103	$10,547	$20	$20,480	20
Blood test: MBS 65070	$16.95	103	$1746	$3	$3390	20
Electrolytes, liver function test, calcium, and phosphate: MBS 66512	$17.70	103	$1823	$4	$3540	20
25 OH, vitamin D: MBS 66833	$30.05	103	$3095	$6	$6010	20
Thyroid‐stimulating hormone: MBS 66716	$25.05	103	$2580	$5	$5010	20
Parathyroid hormone: MBS 66695	$30.50	103	$3142	$6	$6100	20
Weighted average medications cost (3 years)^a^	$1416	34	$47,790	$93	$92,796	21, 22, 23
Office and surgery overheads^b^	27.5% of labor costs		$22,405	$44	$43,506	17
Estimated refracture costs ($2015)						
Hip	$33,781	22	$743,179	$1443	$1,443,067	9, 2
Wrist	$6559	8	$52,471	$102	$101,885	9, 2
Vertebral	$8242	9	$74,181	$144	$144,041	9, 2
Other	$10,458	38	$397,390	$772	$771,631	9, 2

Usual Care Cohort						
Patients	No.					Source [reference no.]
ED MTF patients	416					9

Estimated refracture costs ($2015)	Cost per unit (AUD)	Units utilized (*n*)	Total cost (AUD)	Cost per ED MTF patient (AUD)	Cost per 1000 ED MTF patients ($)	Source
Hip	$33,781	24	$810,741	$1949	$1,948,897	9, 2
Wrist	$6559	6	$39,353	$95	$94,599	9, 2
Vertebral	$8242	15	$123,635	$297	$297,200	9, 2
Other	$10,458	43	$449,678	$1081	$1,080,957	9, 2

JHH = John Hunter Hospital; ED = emergency department; MTF = minimal trauma fracture; AUD = Australian dollars; MBS = Medical Benefits Scheme.

^a^ After allowing for expected 3‐year adherence levels (50% by the end of year 1; 35% by the end of year 2; and 25% by the end of year 3) and initial prescription of medications to 66.66% of patients, we calculated that medication use would be equal to approximately 33% of 103 patients at full adherence (*n* = approximately 34).

^b^ Calculated on labor costs for nursing staff and MBS 110 initial consultation and MBS 116 follow‐up consultation.

### Base case assumptions

- The 20% of FLS Cohort members who responded to JHH FLS invitations all received specialist clinical assessment and the full range of tests described above. This assumption supports a conservative estimate of FLS costs.
- Two‐thirds of FLS Cohort members who responded to JHH FLS invitations received antiresorptive drug therapy. Our study had no specific data on this therapy level and used a finding from a previous study conducted in the same clinic.^21^
- In the absence of observed adherence, antiresorptive (drug) therapy adherence was consistent with that found by Landfeldt and colleagues,^25^ with annual declines to 50% by the end of year 1; 35% by the end of year 2; and 25% by the end of year 3.
- In both cohorts, our data captured all refractures. This assumption is made in the absence of data on other refractures.
- For the 3‐year period, no members of the Usual Care Cohort received any treatments like those administered by the FLS Clinic to the FLS Cohort. This assumption is tested in the sensitivity analysis.

### Uncertainty analysis

Given our limited sample sizes, we estimated the uncertainty around the differences in costs and effect sizes between the FLS and Usual Care cohorts using a Monte Carlo simulation.^26^ Two thousand iterations were conducted using the software package Ersatz^26^ (see Supplemental Materials and Methods for parameter information).

### Sensitivity analysis

Because the base case assumes that the Usual Care Cohort received no post‐fracture preventative care, sensitivity analyses were conducted in consideration of the likelihood that at least some of the Usual Care Cohort did access post‐fracture preventative care and generate costs to the Australian health system.

We modeled three scenarios wherein 5%, 10%, and 20% of the Usual Care Cohort received post‐fracture care commensurate (ie, costing the same per patient) with that received by the JHH FLS Cohort. The percentages were chosen as conservative estimation of the likelihood of post‐fracture care, based on past reports of post‐fracture care in Australia of 30% to 45% of patients receiving at least some level of care.^27^, ^28^, ^29^ For the Usual Care Cohort, we assume that medication adherence rates are the same as for FLS cohorts, as described previously and based on a previous study.^25^

## Results

### Cohorts

Our data on cohorts are limited to the data contained in hospital records. Median ages for the Usual Care and FLS cohorts were, respectively, 77 and 79 years. Females at the Usual Care and FLS cohorts respectively numbered 306 (73.6%) and 388 (75.3%). For both cohorts, Indigenous patients were less than 1%. No data were available on the cohorts’ ethnic makeups. On the basis of postcodes and 2011 Socio Economic Indexes for Areas (SEIFA) (Index of Relative Socio‐economic Advantage and Disadvantage),^30^ we were able to compare socioeconomic disadvantage status of the groups. With this approach, socioeconomic status is rated by decile scores, with higher deciles indicating lower socioeconomic disadvantage within the state of NSW. Among the Usual Care Cohort, 85.5% were in the lower five deciles; the FLS Cohort showed only 45.9% in this range. A significant statistical difference between the distributions of socioeconomic status within the groups was confirmed by a Kruskal‐Wallis equality of population rank test (*p* = 0.0001). However, the simple association between socioeconomic status and clinical incidence rate was not significant. Also, the addition of socioeconomic status as a confounder changed the coefficient for FLS/Usual Care by only 4 points (0.71 to 0.75). Hence socioeconomic status was excluded from the model.

Our data source gave no information on body mass index, fracture risks, or use of glucocorticoids or the prevalence of secondary osteoporosis. With respect to prior treatments for fractures, the FLS Cohort was filtered for previous fracture clinic attendees at recruitment. However, the study was unable to exclude or control for participants who had received previous fracture treatment.

### Base case

Three‐year clinical incidence rates of refractures were found to be 0.212 for the Usual Care Cohort and 0.150 for the FLS Cohort. The calculation for the FLS Cohort includes all its members—including those who did not respond to invitations for free specialist consultations. This is a relative risk (FLS/Usual Care) of 0.71 (0.150–0.212). The FLS Cohort clinical incidence rate was significantly lower (*p* = 0.026) as estimated in a Poisson regression where the outcome variable was number of refractures. Extrapolating these results over 1000 patients gives 212 refractures based on Usual Care Cohort results and 150 refractures based on FLS Cohort results—a reduction of 62.

The total 3‐year cost of managing 1000 patients processed through the JHH FLS was $2,804,378 ($343,753 for conduct of the FLS and $2,460,624 for cost of treatment of the 150 observed refractures), whereas for the Usual Care Cohort, $3,421,653 for cost of treatment of 212 refractures, with no assumed cost for fracture prevention measures (Table 2). For the base case, therefore, the FLS yields an estimated saving of $617,275 over the 3‐year time horizon per 1000 patients processed (Table 1).

**Table 2:** Costs/Savings Fracture Liaison Service (FLS) Versus Usual Care: Base Case and Sensitivity Analysis

	FLS	Base case Usual Care obtain no FLS		Scenario 1 5% of Usual Care obtain FLS		Scenario 2 10% of Usual Care obtain FLS		Scenario 3 20% of Usual Care obtain FLS	
		Usual Care (AUD)	FLS net cost (AUD)	Usual Care (AUD)	FLS net cost (AUD)	Usual Care (AUD)	FLS net cost (AUD)	Usual Care (AUD)	FLS net cost ($)
FLS treatment path	a	b	c (a–b)	d	e (a–d)	f	g (a–f)	h	I (a–h)
FLS assessment of ED records	$38,143	$0	$38,143	$0	$38,143	$0	$38,143	$0	$38,143
FLS contact with patients/GPs	$42,732	$0	$42,732	$0	$42,732	$0	$42,732	$0	$42,732
FLS examination/treatment	$239,860	$0	$239,860	$59,965	$179,895	$119,930	$119,930	$239,860	$0
FLS Follow up	$23,018	$0	$23,018	$5,755	$17,264	$11,509	$11,509	$23,018	$0
Total post‐ED FLS costs	$343,753	$0	$343,753	$65,720	$278,034	$131,439	$212,314	$262,878	$80,875
									
Refracture treatments	$2,460,624	$3,421,653	–$961,029	$3,421,653	–$961,029	$3,421,653	–$961,029	$3,421,653	–$961,029
									
Total per 1000 processed patients	$2,804,378	$3,421,653	–$617,275	$3,487,373	–$682,995	$3,553,092	–$748,715	$3,684,531	–$880,154
Change on base case					10.6%		21.3%		42.6%
									
Refractures per 1000 processed ED patients (*n*)	150	212	–62	212	–62	212	–62	212	–62

AUD = Australian dollars; ED = emergency department; GP = general practitioner.

### Uncertainty analysis

**Figure 2:**
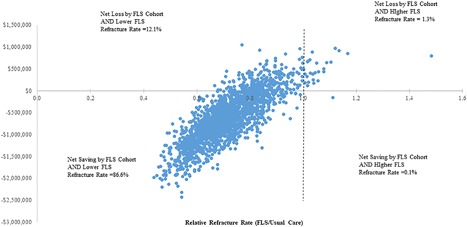
Uncertainty analysis. Net total cost of fracture liaison services by relative refracture rates over 3 years by 1000 processed patients. Monte Carlo simulation: 2000 iterations.

Among 2000 Monte Carlo simulations, 86.6% showed a net saving for the FLS Cohort (cost of FLS − cost of Usual Care) with fewer refractures than Usual Care (ie, relative refracture risk (FLS/Usual Care) less than 1 (Fig. 2) (see Supplemental Material for further results).

### Sensitivity analysis

Scenarios 1, 2, and 3 examine 5%, 10%, and 20% of Usual Care patients receiving a standard of care equivalent to that received by the FLS Cohort at the same cost, while still having the same number of refractures. Results are for net savings after deducting the costs of JHH FLS. For every 1000 patients over 3 years, results were: scenario 1: cost savings of $682,995, a 10.6% increase in savings on the base case; scenario 2: cost savings of $748,715, a 21.3% increase in savings on the base case; scenario 3: cost savings of $880,154, a 42.6% increase in savings on the base case (Table 2).

## Discussion

Although there is increasing acceptance of FLS as an effective means of reducing osteoporotic refracture risk,^31^ implementation of FLS have in part at least been held back by the paucity of observation‐based assessment of their cost and benefit. Previous studies have reported generally positive though variable effects of FLS, with findings generally based on projected or anticipated benefits rather than on observed outcomes and longer time horizons. In a North American study, Solomon and colleagues^11^ found a cost savings of $66,879 ($US 2010) for an FLS clinic compared with Usual Care for every 10,000 treated post‐fracture patients. Using British data, McLellan and colleagues^12^ found cost savings of £21,000 (£GBP 2009) for FLS post‐fracture patients for a hypothetical cohort of 1000 patients, averaging costs across eligible patients who attended and did not attend FLS. Results for both these studies were based on simulation models of comparative lifetime costs and refractures.

Other studies of FLS services have not found base case savings. In a study of an Australian FLS, Cooper and colleagues^32^ modeled costs over 10 years and estimated that compared with Usual Care, an FLS increased per patient cost by $1486 ($AUD 2010). This study averaged FLS costs only over FLS attendees and included repeated annual dual‐energy X‐ray absorptiometry (DXA) scans, lab tests, and medications over 10 years (once a year for the first 4 years and every 3 years thereafter). In a Japanese study, Moriwaki and colleagues^33^ found a base case FLS cost of $3396 ($US 2016) per treated patient. This study was also based on a simulation of lifetime costs and was limited to women who initially had hip fractures.

The present study is not a simulation but rather a micro‐costing of two actual cohorts with real‐life data of refractures, 3 years after initial emergency department presentations. Costs of the FLS Cohort are spread across all members, including those who did not attend the FLS. The study, therefore, presents a fair cost comparison of patients attending two sites, one with an FLS and one without.

In this analysis, we utilized the observed impact of an FLS on refracture risk and compared the health system costs of having a FLS to the usual care situation where there is no FLS. The findings show that over a range of scenarios, varying from the non‐FLS‐processed patients incurring no treatment costs to scenarios where at least some of these “usual care” patients also receive treatment, there is a net cost savings of approximately $617,000 to $880,000 to the Australian health care system for every 1000 patients processed over 3 years.

The base case results, as expressed per 1000 patients over 3 years, can be reexpressed as an estimate of the expected annual savings to the health system. The JHH FLS has operated continually since 2007, and we may conceptualize the Health System realizing delayed annual savings since the end of its third year of existence. The JHH FLS has continually processed about 2000 MTF patients a year.^34^ Because our findings are based on 1000 patients, our annual estimate is doubled. Hence, we estimate an annual savings of $1,234,830 (base case result doubled) to $1,760,308 (scenario 3 doubled).

The current level of adherence rate was based on the findings of Landfeldt and colleagues,^25^ giving a savings of $617,275 per 1000 patients over 3 years. It can be safely assumed that the adherence rate will never be 100%. However, if we test the base case by increasing adherence rates overall for the 3‐year time horizon by a further 15%, 25%, and 35%, the respective savings in the base case decrease to $603,356, $594,077, and $584,797.

The current model assumes that FLS group non‐responders access no similar treatment in the Australian public health system. However, where non‐responders had accessed complete FLS treatment elsewhere within the Australian public health system, the costs to the system will increase, reducing the savings realized through the JHH FLS service. It can be safely assumed that the rate of alternative access among non‐responders is not 100%. However, if we test the base case for treatment rates of 10%, 20%, and 30%, assuming the treatment costs per patient are the same as for those who accessed the FLS, the respective savings to the system decrease to $502,970, $388,664, and $274,358 per 1000 patients over 3 years.

The study has limitations. First, our specific findings are only directly relevant to a comparison of the two hospitals providing data. Our costings are related to the Australian health care system and as such are not directly generalizable to other health care systems where different cost factors operate. However, given that minimal trauma fractures represent a national problem, our findings have qualified pertinence to policy makers considering various options to address this issue.

Second, the costs of refracture management are derived rather than observed. Third, the study was not based on a randomized controlled trial; rather, it utilized an observational study of two similar‐sized cohorts of generally well‐matched patients followed from the beginning of an intervention period.^9^ A preferable research design would have observed both groups before the commencement of the intervention for the FLS Cohort (ie, “difference in differences analysis”^35^). This would have allowed observation and modeling of general trends resulting from factors affecting both cohorts, not accounted for in our data.^35^ Fourth, sensitivity analysis could be applied more broadly, considering for example, the costs of possible preventative treatments accessed elsewhere by members of the FLS Cohort who did not respond to offers of consultations from the FLS.

Other limitations bias our estimates conservatively, ie, underestimate FLS savings. Although a previous analysis of the same data^9^ used a competing risk model where those who died were no longer able to have a fracture, in this economic analysis, all patients in the cohorts were assumed to survive for the entire 3‐year follow‐up and to accrue medication costs.^9^ Our assumed medication adherence rates, although based on evidence, would likely have been different had they been observed.^36^

Also, the limitations of our data meant that we could not adjust our findings for bone mineral density, prior fractures, ethnicity, prior fracture treatments, and use of glucocorticoids. Although the coding practices in the two hospitals were the same, as with all studies that utilize hospital recorded data, there is a risk of quality variation that could affect accuracy.

Further, our time horizon of 3 years excludes costs that may arise in the longer term. For example, because of their lack of preventative FLS‐type treatment, the Usual Care Cohort can be expected to have a sequelae of other morbidities and admissions in later life. No costs associated with such sequelae are included in our model, underestimating the costs for Usual Care. A further limitation is our exclusion of health care costs for services to MTF patients provided by general practitioners. Because we had no data, we were unable to account for GP costs to the health system. Also, in this study, we did not undertake a cost utility analysis and were unable to consider the broader societal costs or the likely effect on quality‐of‐life measures of osteoporotic fractures.

Despite the limitations in the research design, the study retains strengths. First, the modeling is a direct comparison of the refracture treatment of two cohorts, over the same time period, in the same state of the same country, from hospitals of similar size and function.^9^ As best as can be ascertained, the control group was not directly offered immediate specialist attention in an FLS. Effectiveness studies have previously not compared groups of fracture patients with and without exposure to offers of FLS services at hospital of their initial attendance. Further, the study design contains documented observations of refractures for both groups, identifying fracture types and thereby not relying on patient‐provided data with reliability issues.

From the perspective of Australian public health services, the evidence of this study is that an FLS generates an important gain by reducing avoidable downstream costs. Our finding of a rounded net positive effect of approximately $617,000 to $880,000 over 3 years per 1000 patients processed is likely to be an underestimate. Because the JHH FLS services approximately 1000 patients per 6 months, our estimated saving for the JHH clinic would annually accrue $1,234,000 to $1,760,000. Wide rollout of FLS has the potential to realize significant resource savings for Australian health care services.

## Disclosures

All authors state that they have no conflicts of interest.

## Supporting information

Supporting Data S1.
