# Supplementary material for: The Costs of Confronting Osteoporosis: Cost Study of an Australian Fracture Liaison Service
Source: JBMR Plus. 2018 Apr 18;3(1):56–63. doi: 10.1002/jbm4.10046 (PMC6339551; doi:10.1002/jbm4.10046)
Supplement: Supplementary file 1 — Supporting Data S1. [file JBM4-3-56-s001.docx]

**Supplementary Materials**

**Uncertainty Analysis**

Uncertainty analysis was performed by applying distributions (Supplementary Table 1) first, for variables with respect concerned with attendance at the FLS clinic:

1) percentage of patients contacted by the FLS who attended the clinic

2) percentage of attendees undertaking second follow up visits

3) percentage of FLS attendees requiring anti-resorptive (medication).

Beta distributions were chosen, as appropriate for continuous variables, with set means (observed) and standard deviations, giving values that would not give values below 0% or above 100%^1^.

Note that the standard deviations of all three inputs relating to 25% of their observed values. This was a subjective choice in the absence of further evidence.

Distributions of re-fractures for both FLS and Usual Care were set using the Poisson distribution, again with observed values as the means. Poisson distributions were chosen as the re-fractures are regarded as ‘event’ or ‘count’ data. No standard deviations are required with Poisson distributions.

Monte Carlo simulations were conducted in Ersatz software^2^. The numbers of iterations was set at 2,000 to give robust results, the Ersatz Guide recommending usually at least 2,000.

Model outputs included were total re-fracture numbers and relative re-fracture rates (FLS Cohort re-fracture rate/Usual Care re-fracture rate) and net costs/savings of the FLS Cohort (FLS Cohort costs – Usual Care Cohort costs). Supplementary Table 2 shows output statistics with a mean cost saving for the FLS Cohort of $619,925, just higher than the observed value of $617,275. Collation showed that FLS cost losses (i.e. where net costs of FLS total were greater than $0) occurred in 13.4% of the 2,000 iterations. Savings for FLS occurred in 86.6% of the 2,000 iterations. In the main article, Fig. 2 presents a scatter plot of iteration results for FLS Cohort net costs by relative re-fracture rates.

Monte Carlo runs were also conducted with standard deviations for variables 1), 2) and 3) set at 35% and 50% of observed values. Respective percentages of cost losses for FLS (i.e. FLS costing more than usual care) occurred in also 13.7% and 14.6% of each set of 2,000 iterations, showing only minor changes in the percentages of losses with increasing standard deviations.

**Supplementary Table 1** Uncertainty Parameters and Distributions

| Parameter | |  | Data | Distribution |
| --- | --- | --- | --- | --- |
| FLS Cohort | |  |  |  |
|  | Attendance and treatment (%(standard Deviation*)) | |  |  |
|  |  | Contacted JHH ED MIF patients attending JHH FLS (FLS Attendees) | 20% (5%) | Beta |
|  |  | Percentage of FLS attendees with follow up visit | 55.3% (14%) | Beta |
|  |  | Percentage of FLS attendees receiving anti-resportive therapy | 66.6% (17%) | Beta |
|  | Re-fractures (no.)** | |  |  |
|  |  | Hip | 22 | Poisson |
|  |  | Wrist | 8 | Poisson |
|  |  | Vertebrae | 9 | Poisson |
|  |  | Other | 38 | Poisson |
| Usual Care Cohort | | |  |  |
|  | Re-fractures (no.)** | |  |  |
|  |  | Hip | 24 | Poisson |
|  |  | Wrist | 6 | Poisson |
|  |  | Vertebrae | 15 | Poisson |
|  |  | Other | 43 | Poisson |
|  |  |  |  |  |
| *Standard deviations arbitrarily set at 25% of observed values, the inputted mean values in the modelling  ** Numbers of re-fractures counted in the observed data, which is converted to re-fracture rates. | | | |  |

**Supplementary Table 2** Costs - FLS vs Usual Care: Uncertainty Analysis

| **Base Case Uncertainty Analysis Results** | |  |  |  |  |  |  |  |
| --- | --- | --- | --- | --- | --- | --- | --- | --- |
|  |  | Median | Mean | Standard Deviation | Lowest Value | Highest Value | Low CI 95% | High CI 95% |
| Re-fractures | |  |  |  |  |  |  |  |
|  | Relative Re-fracture Risk Rate (FLS/UC) | 0.71 | 0.72 | 0.11 | 0.44 | 1.48 | 0.52 | 0.96 |
|  | Net Refractures (n) FLS - UC | -61 | -62 | 28 | -150 | 64 | -117 | -6 |
| Costs |  |  |  |  |  |  |  |  |
|  | Total Post-ED FLS Costs | $338,146 | $342,664 | $50,988 | $220,435 | $562,354 | $257,610 | $455,800 |
|  | Net Re-fracture Treatments (FLS-UC) | -$970,780 | -$962,590 | $547,121 | -$2,735,390 | $649,359 | -$2,088,298 | $81,365 |
|  | **Total Net Costs/Savings ($)** | **-$627,019** | **-$619,925** | **$549,529** | **-$2,434,229** | **$1,051,435** | **-$1,731,182** | **$430,995** |
| 2,000 iterations | |  |  |  |  |  |  |  |
| All results as per 1,000 patients | |  |  |  |  |  |  |  |
| Costs >$0 indicate FLS net cost | |  |  |  |  |  |  |  |
| Costs <$0 indicate FLS net saving | |  |  |  |  |  |  |  |
|  | Iterations where Total Net Savings due to FLS > $0=86.6% | |  |  |  |  |  |  |

**References**

1. Barendregt J. Ersatz User Guide. Brisbane: Epigear International Pty. Ltd; 2012.

2. Barendregt J. Ersatz. Brisbane, Australia: Epigear International; 2012. p. 1.2.
